# High Impact Exercise Improves Bone Microstructure and Strength in Growing Rats

**DOI:** 10.1038/s41598-019-49432-2

**Published:** 2019-09-11

**Authors:** Tanvir Mustafy, Irène Londono, Florina Moldovan, Isabelle Villemure

**Affiliations:** 1 Department of Mechanical Engineering, École Polytechnique of Montréal, P.O. Box 6079, Station Centre-Ville, Montréal, Québec H3C 3A7 Canada; 2 Sainte-Justine University Hospital Center, 3175 Côte-Sainte-Catherine Rd., Montréal, Québec H3T 1C5 Canada; 3 Department of Stomatology, Faculty of Dentistry, Université de Montréal, Montreal, P.O. Box 6128, Station Centre-Ville, Montréal, Québec H3C 3J7 Canada

**Keywords:** Biomedical engineering, Bone

## Abstract

Physical activity is beneficial for skeletal development. However, impact sports during adolescence, leading to bone growth retardation and/or bone quality improvement, remains unexplained. This study investigated the effects of *in vivo* low (LI), medium (MI), and high (HI) impact loadings applied during puberty on bone growth, morphometry and biomechanics using a rat model. 4-week old rats (n = 30) were divided into control, sham, LI, MI, and HI groups. The impact was applied on the right tibiae, 5 days/week for 8 weeks mimicking walking (450 µε), uphill running (850 µε) and jumping (1250 µε) conditions. Trabecular and cortical parameters were determined by micro-CT, bone growth rate by calcein labeling and toluidine blue staining followed by histomorphometry. Bio-mechanical properties were evaluated from bending tests. HI group reduced rat body weight and food consumption compared to shams. Bone growth rate also decreased in MI and HI groups despite developing thicker hypertrophic and proliferative zone heights. HI group showed significant increment in bone mineral density, trabecular thickness, cortical and total surface area. Ultimate load and stiffness were also increased in MI and HI groups. We conclude that impact loading during adolescence reduces bone growth moderately but improves bone quality and biomechanics at the end of the growing period.

## Introduction

A fundamental tenet of bone biomechanics is the adaptation phenomenon of bone microstructure under regularly applied mechanical loading^1,2^. It has been hypothesized by “Wolff’s law”^3^ that this dynamic adaptive process, termed bone remodeling, occurs in bone mass and architecture due to stimuli obtained from its mechanical environment^4,5^. Bones can receive stimuli in the form of mechanical loading resulting from various intensities in physical activities and sports. Ground reaction forces, which generate stresses and strains on our weight-bearing bones, are determinant factors of bone remodeling. They are greater when we move faster and/or more intensively, hence vary according to the type of physical activities^6^. Low impact sports such as swimming, can produce lower ground reaction forces than high impact sports such as gymnastics or cross-country running^7,8^. A regular and sufficient amount of impact loading can prove to be very effective for bones at all ages^9^.

Well-controlled and physiologic mechanical loading models are essential to successfully define and identify the anabolic and catabolic mechanisms involved in bone remodeling. To date, many loading models have been implemented in different animal studies, ranging from whole body rodent vibration, exercise models, and *in vivo* bone loading models such as tibial bending, ulnar and tibial axial loading^10–14^. Compared to other models, the tibial compression model has the potential to generate cortical and trabecular bone adaptation under applied mechanical load^15,16^. Axial loading in mouse tibia has been used for several years to investigate the effects of loading as a function of age^17,18^, sex^19^, disease^20^ and strain level^16^. However, still, there is a lack of data on the loading mechanism and effects of non-invasive loading in bone formation for the rat tibial axial compression model.

Adolescence is a dynamic period for bone growth and development^21^. In this period, regular impact loading in sufficient amount can ensure a proper bone accrual and also contribute to building up a strong skeleton^22^. It has been reported that^23^ performing a high impact physical activity, such as jumping, can effectively contribute to improving the hip bone strength in adolescents^24^. There are a few clinical and animal studies investigating the effects of impact loading on adolescent growth, but results are inconsistent. In some published clinical studies^25,26^, researchers have not provided a clear, distinct separation between the nutritional and mechanical factors. The studies expressed the physical activities in hours per week whereas the intensity of the activity (repetitions, peak load, and frequency) have not been considered. As a result, it becomes challenging to infer about the isolated effect of impact loading on the growing bones from these studies. In animal studies, the effects of exercise on long bone growth are also inconsistent, resulting in either no change^27^, minimal change^28^, or significant reduction^29,30^ in growth, measured as changes in long bone length. Hence, if any loading induced changes occur during the adolescence and whether they modify the bone microstructure are clinically relevant questions which remain still unresolved. We hypothesize that bone morphometry and biomechanics can be improved, but bone growth would remain unaffected under the controlled impact loading applied during the adolescence period. Moreover, among three different impact loading levels, we suggest that the higher impact intensity influences more importantly bone morphometry and biomechanics. This study aimed to use an animal model (rat tibia) to investigate the effects of well controlled *in vivo* low, medium and high impact loadings applied during puberty on bone growth, morphometry and biomechanics.

## Results

### Body weight and food intake

**Figure 1:**
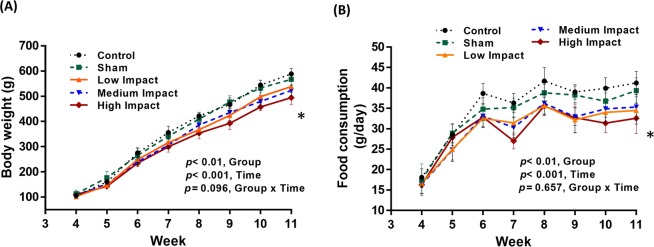
Rat body weight (g) and food consumption (g/day) during experimental period. (**A**) ANOVA test (general linear model) was performed to determine time effects, group effects, and their interaction on body weight. (**B**) ANOVA test (general linear model) was performed to determine time effects, group effects, and their interaction on food consumption. *N* = *6 rats per group (mean value* ± SD). *p < *0.05: significant compared to shams*.

The control and sham groups gained body weight whereas the HI group lost 13–16% of body weight compared to the shams at the end of the loading period (Fig. 1A). Food intake was depressed by about 17–20% (Fig. 1B) in HI group at the end of the study compared to the shams. However, no significant difference in the mean body weights and food consumption were found among the three impact groups during the loading period. A time effect (weight gain and increase in caloric consumption) was observed in rats as they were in their growing phase. A group effect was also noticed, but no effects of group/time interaction were found (Fig. 1A,B). Also, no evidence of swelling or limb-use impairment, and no loss of hair was noticed in the animals throughout the experiment.

### Bone growth rate and tibial length

**Figure 2:**
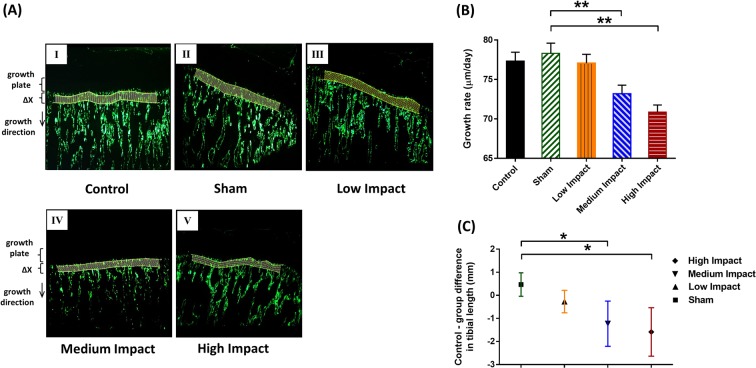
Bone growth rates (µm/day) and longitudinal tibial lengths (mm). (**A**) 2.5x magnified microscopic images of the tibial metaphysis labeled twice with calcein and representative images of tibiae for control, sham, LI, MI and HI groups (I–V). Bone growth (ΔX, μm) measured as the mean distance between the two calcein lines, which were modeled as splines and divided by the time interval (3 days) between the two applied injections. (**B**) Bone growth rates (μm/day) of rat proximal tibiae for control, sham, LI, MI and HI groups. (**C**)Relative (control minus individual group) gross tibial length (mm) of the tibiae. MI and HI groups exhibited approximately three and four times reduction in tibial length difference. *N* = *6 rats per group (mean value* ± SD). *p < 0.05 and **p < *0.01: significant compared to shams*.

Both HI and MI groups exhibited a reduction in proximal tibial growth rate (Fig. 2B), resulting in 8.7% and 5.6% decrease for HI and MI groups, respectively, compared to shams (Fig. 2B). Relative gross tibial length (value for control rats minus rats from other groups) exhibited significant differences for HI and MI groups (Fig. 2C). No significant difference was observed between control and sham groups for both growth rates (Fig. 2B) and relative tibial lengths (Fig. 2C).

### Growth plate histomorphometry

**Figure 3:**
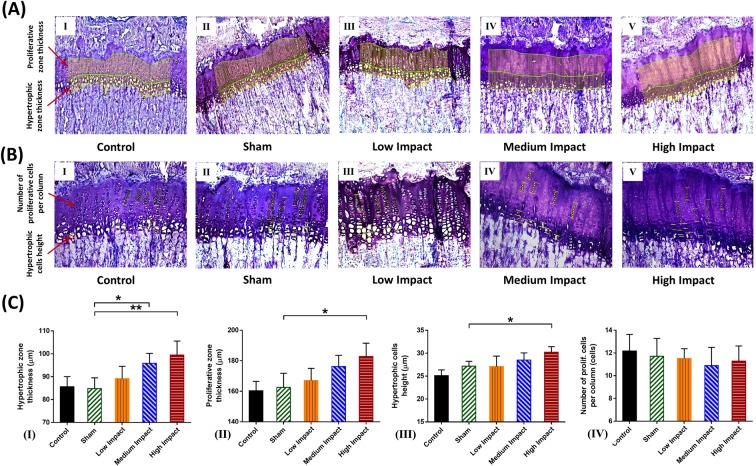
Growth plate histomorphometric parameters for control, sham, LI, MI and HI tibiae. (**A**) Growth plate section embedded in MMA and stained with toluidine blue (10x). Evaluation of the hypertrophic and proliferative zonal thicknesses (µm) for control, sham, LI, MI and HI groups (I–V). (**B**) Growth plate section embedded in MMA and stained with toluidine blue (20x). Evaluation of the hypertrophic cell height (µm) and number of proliferative cells per column (cells) for control, sham, LI, MI and HI groups (I–V). (**C**) Growth plate histomorphometry measurements of rat proximal tibiae for control, sham, LI, MI and HI groups (I–V). *N* = *6 rats per group (mean value* ± SD). *p < 0.05 and **p < *0.01: significant compared to sham*.

Hypertrophic (HZ) and proliferative (PZ) zone thicknesses (Fig. 3A), as well as the number of proliferative cells per column and hypertrophic cells height (Fig. 3B), have been evaluated for all the experimental groups. MI and HI group exhibited significantly thicker (13% and 17%, respectively) HZ thickness compared to the sham group (Fig. 3C–I). Moreover, PZ thickness also increased in HI groups (12%) compared to shams (Fig. 3C–II). Hypertrophic cell heights were also increased by 12% in HI group compared to the shams (Fig. 3C–III). The number of proliferative chondrocytes per column was similar for all groups (Fig. 3C–IV). Control vs. sham groups showed no significant difference in growth plate histomorphometric parameters (Fig. 3C).

### Trabecular and cortical bone architecture

The effects of impact loading exercise on bone microstructure were assessed by comparing the impact groups with the shams after 4 and 8 weeks of repeated loading regime. For trabecular bone, the HI group showed an increase in BV/TV and a decrease in Tb.Sp (Table 1) after 4 weeks of loading. For cortical bone, Ct.Ar was increased in HI group at this time point (Table 2). After 8 weeks of loading, a significant increase was found in BMD and Tb.Th for both HI and MI groups (Table 1). However, only HI group showed a significant increment in BV/TV and a significant decrement in Tb.Sp at this time point (Table 1). For the cortical bone architecture, both HI and MI exhibited an increased Ps.Pm and Ct.Ar and a decreased Ec.Pm (Table 2) after 8 weeks. However, only HI group showed an increased Tt.Ar and Ct.Th and a decreased Ma.Ar at this time point (Table 2).

**Table 1:** ANOVA test with Tukey’s multiple comparisons for the trabecular microarchitecture of the right proximal tibial metaphysis in control, sham, LI, MI and HI groups of rats after 4 weeks and 8 weeks of loading regime.

Parameters/Groups	Control	Sham	L.I.	M.I.	H.I.
***4 weeks of loading***					
BMD (g.cm^−3^)	0.19 ± 0.01	0.18 ± 0.01	0.19 ± 0.02	0.19 ± 0.02	0.22 ± 0.02
BV/TV (%)*	21.7 ± 3.11	21.2 ± 1.13	22.1 ± 2.83	22.8 ± 2.19	**23.9 ± 1.26** ^**α**^
Tb.Th (mm)	0.08 ± 0.01	0.08 ± 0.02	0.09 ± 0.01	0.09 ± 0.02	0.09 ± 0.01
Tb.N (mm^−1^)	2.37 ± 0.11	2.32 ± 0.12	2.56 ± 0.12	2.44 ± 0.09	2.43 ± 0.09
Tb.Sp (mm)*	0.79 ± 0.09	0.77 ± 0.13	0.75 ± 0.13	0.76 ± 0.12	**0.71 ± 0.11** ^**α**^
Conn.Dn (mm^−3^)	174 ± 30.1	169 ± 33.1	175 ± 33.2	170 ± 36.3	162 ± 36.3
***8 weeks of loading***					
BMD (g.cm^−3^)*	0.22 ± 0.01	0.23 ± 0.01	0.24 ± 0.01	**0.25 ± 0.01** ^**α**^	**0.27 ± 0.01** ^**α**^
BV/TV (%)*	23.7 ± 2.79	23.3 ± 3.12	24.3 ± 1.75	26.9 ± 1.69	**28.7 ± 2.55** ^**α**^
Tb.Th (mm)*	0.09 ± 0.01	0.09 ± 0.01	0.10 ± 0.02	**0.11 ± 0.01** ^**α**^	**0.11 ± 0.02** ^**α**^
Tb.N (mm^−1^)	2.92 ± 0.15	2.81 ± 0.15	3.15 ± 0.17	3.05 ± 0.09	2.98 ± 0.08
Tb.Sp (mm)*	0.78 ± 0.07	0.77 ± 0.06	0.66 ± 0.07	0.69 ± 0.07	**0.59 ± 0.05** ^**α**^
Conn.Dn (mm^−3^)	157 ± 22.3	161 ± 22.1	159 ± 25.9	156 ± 27.3	149 ± 28.3

Values are expressed as Mean ± SD, N = 6/group. In the parameter column, *indicates a significant effect (*p* < 0.05) from a one-way ANOVA with Tukey’s multiple comparisons test. When there was a significant effect, Tukey’s post-hoc pairwise comparisons evaluated whether the sham group was significantly different compared to the other groups [a bold value and ‘α’ indicate a significant difference versus sham group]. Abbreviations: BMD, bone mineral density; BV/TV, bone volume fraction; Tb.Th, trabecular thickness; Tb.N, trabecular number; Tb.Sp, trabecular spacing; Conn.Dn, connectivity density.

**Table 2:** ANOVA test with Tukey’s multiple comparisons for the cortical microarchitecture of the right tibial mid-diaphysis in control, sham, LI, MI and HI groups of rats after 4 weeks and 8 weeks of loading regime.

Parameters/Groups	Control	Sham	L.I.	M.I.	H.I.
***4 weeks of loading***					
TMD (gm.cm^−3^)	0.87 ± 0.06	0.96 ± 0.06	0.91 ± 0.05	1.05 ± 0.08	1.08 ± 0.08
Tt.Ar (mm^2^)	5.73 ± 0.97	5.84 ± 1.05	5.89 ± 0.88	6.12 ± 1.17	6.14 ± 0.98
Ct.Ar (mm^2^)*	4.26 ± 0.61	4.12 ± 0.51	4.32 ± 0.59	4.89 ± 0.72	**5.41 ± 0.72** ^**α**^
Ct.Th (mm)	0.51 ± 0.02	0.54 ± 0.02	0.54 ± 0.02	0.58 ± 0.02	0.62 ± 0.02
Ps.Pm (mm)	9.56 ± 0.37	9.12 ± 0.47	9.72 ± 0.71	9.94 ± 0.59	10.1 ± 0.51
Ec.Pm (mm)	4.73 ± 0.44	4.82 ± 0.33	4.93 ± 0.33	4.95 ± 0.47	4.75 ± 0.22
Ma.Ar (mm^2^)	1.62 ± 0.05	1.73 ± 0.06	1.62 ± 0.06	1.71 ± 0.08	1.69 ± 0.07
Ecc	0.63 ± 0.03	0.63 ± 0.03	0.61 ± 0.06	0.64 ± 0.05	0.63 ± 0.04
***8 weeks of loading***					
TMD (gm.cm^−3^)	1.08 ± 0.08	1.04 ± 0.09	1.09 ± 0.07	1.15 ± 0.09	1.18 ± 0.09
Tt.Ar (mm^2^)*	8.17 ± 1.11	7.92 ± 0.93	8.33 ± 1.03	8.89 ± 0.94	**9.24 ± 1.03** ^**α**^
Ct.Ar (mm^2^)*	6.12 ± 0.83	6.33 ± 0.88	6.23 ± 0.72	**7.38 ± 0.79** ^**α**^	**7.83 ± 0.82** ^**α**^
Ct.Th (mm)*	0.66 ± 0.02	0.62 ± 0.03	0.63 ± 0.02	0.67 ± 0.02	**0.69 ± 0.02** ^**α**^
Ps.Pm (mm)*	13.5 ± 0.47	13.8 ± 0.62	13.2 ± 0.57	**14.9 ± 0.61** ^**α**^	**15.5 ± 0.67** ^**α**^
Ec.Pm (mm)*	6.43 ± 0.49	6.72 ± 0.48	6.34 ± 0.43	**5.94 ± 0.42** ^**α**^	**5.83 ± 0.42** ^**α**^
Ma.Ar (mm^2^)*	1.92 ± 0.07	1.85 ± 0.08	1.83 ± 0.07	1.78 ± 0.09	**1.73 ± 0.09** ^**α**^
Ecc	0.78 ± 0.02	0.79 ± 0.04	0.79 ± 0.04	0.81 ± 0.04	0.78 ± 0.05

Values are expressed as Mean ± SD, N = 6/group. In the parameter column, *indicates a significant effect (*p* < 0.05) from a one-way ANOVA with Tukey’s multiple comparisons test. When there was a significant effect, Tukey’s post-hoc pairwise comparisons evaluated whether the sham group was significantly different compared to the other groups [a bold value and ‘α’ indicate a significant difference versus sham group]. Abbreviations: TMD, tissue mineral density; Tt.Ar, cross-sectional area inside the periosteal envelope; Ct.Ar, cortical bone area; Ct.Th, cortical thickness; Ps.Pm, periosteum perimeter; Ec.Pm, endocortical perimeter; Ma.Ar, medullary area; Ecc, mean eccentricity.

### Tibial mechanical properties

From three-point bending tests, significant differences were found among the groups for some structural and intrinsic mechanical properties (Table 3). The mean ultimate load, as well as the mean ultimate stress were found higher in the MI and HI groups compared to the sham animals (Table 3). Energy to failure load and stiffness were also increased in MI and HI groups compared to shams (Table 3). Finally, failure stress was found to be significantly lower in the MI and HI groups compared to the sham group (Table 3).

**Table 3:** ANOVA test with Tukey’s multiple comparisons for structural and intrinsic mechanical properties of the right tibiae from control, sham, LI, MI and HI groups of rats derived from three-point bending tests of the mid-diaphysis.

Parameters/Groups	Control	Sham	L.I.	M.I.	H.I.
***Structural mechanical properties***					
Ultimate load, F_ult_ (N)*	84.9 ± 13.3	80.1 ± 11.2	89.4 ± 9.22	**93.5 ± 7.43** ^**α**^	**97.0 ± 8.16** ^**α**^
Failure load, F_fail_ (N)	73.8 ± 11.6	67.3 ± 10.3	65.3 ± 10.6	63.4 ± 9.44	64.6 ± 9.82
Stiffness, k (N/mm)*	123 ± 18.7	113 ± 16.8	124 ± 15.8	**135 ± 17.6** ^**α**^	**142 ± 15.8** ^**α**^
Energy to ultimate load (mJ)	39.3 ± 5.7	35.3 ± 6.11	41.4 ± 6.82	42.2 ± 7.03	44.5 ± 7.26
Energy to failure load (mJ)*	55.2 ± 17.3	63.5 ± 15.2	77.6 ± 18.4	**81.7 ± 14.8** ^**α**^	**79.8 ± 18.9** ^**α**^
***Intrinsic mechanical properties***					
Ultimate stress, σ_ult_ (MPa)*	294 ± 44.5	286 ± 37.2	308 ± 52.1	**319 ± 49.2** ^**α**^	**316 ± 38.2** ^**α**^
Failure stress, σ_fail_ (MPa)*	223 ± 36.2	216 ± 21.2	187 ± 30.3	**184 ± 26.2** ^**α**^	**186 ± 22.4** ^**α**^
Young’s modulus, E (GPa)	10.9 ± 1.22	10.5 ± 1.46	11.2 ± 1.31	11.8 ± 1.01	12.3 ± 1.06
Energy to ultimate stress (mJ/mm^3^)	3.46 ± 1.11	2.71 ± 0.82	2.56 ± 0.96	2.89 ± 1.21	2.95 ± 0.85
Energy to failure stress (mJ/mm^3^)	8.37 ± 3.46	11.4 ± 4.17	11.7 ± 4.61	12.2 ± 3.19	11.9 ± 4.89

Values are expressed as Mean ± SD, N = 6/group. In the parameter column, *indicates a significant effect (*p* < 0.05) from a one-way ANOVA with Tukey’s multiple comparisons test. When there was a significant effect, Tukey’s post-hoc pairwise comparisons evaluated whether the sham group was significantly different compared to the other groups [a bold value and ‘α’ indicate a significant difference versus sham group].

## Discussion

In this study, the effects of eight weeks controlled impact loading during the adolescence on bone growth, quality and mechanics have been investigated using a rat tibial compression loading model. Our findings could be used as a basis for future investigation on the impact loading effects during the adolescence for finding a suitable loading protocol which would be beneficial for the overall bone microstructure during the growing period.

### High impact loading triggers decreased body weight coupled with a reduced caloric consumption

Body weight was maximum for the control group followed by the shams and other impact groups at the end of the loading period (Fig. 1A). The body weight in HI group was decreased significantly compared to shams after 8 weeks of loading regime (Fig. 1A). Interestingly, food consumption was simultaneously reduced for the same group (HI) at the end of the study (Fig. 1B). The food consumption is generally dependent on the energy expenditure and so is the change in body weight^31^. Our findings showed that the HI group has less body weight and reduced food intake despite receiving the maximum intensity of the exercise regime. Part of this weight loss could be ascribed to the decreased appetite of the impact groups^32^, which was evidenced by a significantly lower caloric intake for the HI group (Fig. 1B). Another intriguing fact was the transitory fatigue of the exercised rats. We observed reduced activity limited to less than seven minutes after the forced loading regime. The rats continued their regular cage activity shortly after this phenomenon. It is suggested that the forced exercise can influence the levels of stress hormones and behavior of the animals which may lead to a reduction in caloric intake in the rats^33,34^. Moreover, bone osteocytes were shown to be sensitive to short term high-impact dynamic loading conditions^35^. It has also been reported that body weight reduction can activate a sensor dependent on osteocytes, which eventually diminishes caloric intake in the rodents^35–37^. The observed reduced body weight and caloric intake for the high impact group could have resulted from this phenomenon.

Our findings are supportive of other studies on adult animal models. Reduced body weight has been reported in trained animals by Jones *et al*.^38^ and Huang *et al*.^31^, after 15 and 8 weeks of exercise period in adult rats, respectively. Moreover, simultaneously reduced body weight and reduced caloric consumption have been reported for adult running rats by Crew *et al*.^39^, and treadmill exercised in post pubertal rats by Tisuji *et al*.^40^ and by Pitts and Bull^32^.

### Medium and high impact loadings decrease longitudinal bone growth despite developing thicker HZ and PZ heights

Both MI and HI groups showed reduced bone growth rates at the proximal metaphysis compared to shams after 8 weeks of loading (Fig. 2B). This phenomenon eventually resulted in significant longitudinal growth retardation for the same two groups (Fig. 2C); it contradicts our hypothesis that longitudinal bone growth rate would remain unaffected under the impact loadings. Some noticeable histomorphometric changes were also concomitant along with this growth retardation. These changes include increased hypertrophic and proliferative zone thicknesses and hypertrophic cell heights (Fig. 3C).

The relationship between applied compression and longitudinal bone growth rate proposed by Hueter-Volkmann law states that increased compression reduces bone growth rate whereas reduced compression increases it^41,42^. Moreover, large compressive loads can lead to retardation of bone growth or even cease completely the bone growth^42–45^. Our findings are also consistent with other studies^46–48^, where rat ulna longitudinal growth was decreased by compressive loading in adolescence.

Bone growth rate is generally correlated to the overall growth plate thickness^7,49^. Moreover, it is considered to be linearly correlated with the PZ^7,31^ and HZ^27,50^ thickness. Hence, the thicker HZ and PZ heights of the MI and HI group were expected to result in elongated bone length. Conversely, bone growth in MI and HI group was depressed even after thickening of growth plates. Previous studies have also reported thicker growth plates under excessive loadings. However, these studies related this phenomenon with dyschondroplasia (osteochondrosis)^46,51^. Osteochondroses are considered to be disorders of primary and secondary growth centers, or lesions at the apophyseal or epiphyseal growth areas of bones^52^. Active young athletes are prone to osteochondroses^52^, although it is not considered to be a permanent disability for diagnosed patients^53^. In most cases, conservative treatment for such symptoms includes sufficiently long rest^52^. In one form of such condition known as Scheuermann’s disease^54^, regular physical exercises are even recommended. In our study, the rats have been sacrificed immediately after the repeated loading regime. So, it remains unclear whether a sufficient amount of unloading period would affect the growth plate thickness and change it accordingly or not.

The change in longitudinal bone growth could also be associated with the caloric intake and body weight of the rodents. It has been reported that the reduction of body weight due to reduced caloric intake can affect cell production in the proliferative zone in a negative manner^55^, which can eventually slow down longitudinal bone growth. Our observations can be compared with the studies using rat^56,57^ and swine^55^ models, where a reduction in body weight coupled with reduction in longitudinal bone growth has been observed. Our overall findings indicate that the generalized claim of the linear relationship between bone growth and growth plate height^7,58^ might not always be implemented. However, our findings are supportive of other studies^7,46,47^, where a contrary relationship between the growth rate and height of the growth plate was also observed.

### Changes in trabecular bone microstructure are time as well as impact level dependent

Compared to the MI group, the HI group showed load adaptive changes in trabecular microstructure at an earlier (after 4 weeks) period and affecting more structural parameters (Table 1). BMD was significantly increased in both MI and HI groups (Table 1) at the end of loading period. For healthy bone structure, bone mineral content shows an increasing trend during the adolescent period^59^. Also, higher loading intensity is generally associated with an increased BMD^60^. In fact, BMD in athletes is elevated under high impact training conditions^61^. The increase of BMD in our study could be related to hormones triggering mechanisms. Indeed, an increased BMD is controlled by a decreased parathyroid hormone response coupled with an increased calcitonin response^62,63^, both of which take time to react under favorable loading conditions. Hence, this could explain the significant increment of BMD assessed after 8 weeks instead of 4 weeks of loading. In similar studies, where adult rats have been tested for repetitive jumping exercise^64^ or treadmill running exercise^65^, an increased BMD was observed in the loaded limbs.

The BV/TV was also found to significantly increase in the exercised tibiae both after 4 and 8 weeks of loading in the HI group. For a healthy growing bone, an elevated BV/TV is generally correlated with an increased BMD^66^, as found in this study. The significant BV/TV in HI group after 8 weeks of loading can be explained with the increased BMD for the same group (Table 1). However, the significant increase after 4 weeks of exercise (without simultaneous BMD increase) could be related to triggering of bone metabolism under high impact loading^61^. Indeed, it has been reported that under controlled loading scenarios, the trabecular structure responds positively through diffusion and active transport of metabolites within the entire microstructure^59,67^. So, it could be possible that HI loading has accelerated the diffusion and transportation process of the metabolites at an earlier stage and thus elevated the BV/TV in the exercised limb accordingly. Other studies support our findings in growing rats, where swimming exercise was found to significantly increase BV/TV^68,69^. Also, another study reported a greater BV/TV in the growing rat tibiae after 8-weeks of free fall exercise routine^70^. The observed increment in Tb.Th during the loading period is a part of normal bone development^71^. However, a significant increment in the loaded limbs (compared to shams) indicated an additional improvement in trabecular structure under impact loading conditions. The significant reduction in Tb.Sp is often considered as the concomitant increase with BV/TV and Tb.Th^72^. The significant reduction in Tb.Sp indicates the occurrence of a loading induced bone gain through increased connectivity and gradual thickening of the trabecular structure^73^. The significant change in Tb.Sp in HI group at an earlier stage can be associated with the significant increase in BV/TV from the same group (Table 1). Our data are supportive of previous findings where an increased Tb.Th was reported in the loaded tibiae of 10-week old adult mice^74^, as well as with a decreased Tb.Sp reported in loaded tibiae of both growing and adult mice^15^.

### Medium and high impact loadings benefit the cortical bone morphometry in the diaphysis, leading to significantly improved structural- and tissue-level bending mechanical properties

MI and HI loadings significantly affected tibial diaphysis, modifying both its cortical microstructure and its mechanical properties (Tables 2 and 3); it supports the hypothesis that bone morphometry and biomechanics are improved by impact loadings and that higher impact intensity has greater positive influence on bone morphometry and biomechanics. Indeed, stiffness was significantly increased in these groups compared to the sham group (Table 3). This improved stiffness eventually triggered the bones to reach a significantly higher ultimate load (Table 3). Interestingly, MI and HI groups exhibited significantly increased cortical area, simultaneously coupled with periosteal perimeter expansion and endocortical perimeter reduction (Table 2) after 8 weeks of loading regime. The medullary area (Ma.Ar) in the HI group also decreased significantly (Table 2). Cortical bone area at the mid-diaphysis and the corresponding ultimate load (F_ult_) are highly correlated to each other^75,76^. Hence, the increased ultimate loads for MI and HI groups can be justified from their significantly increased cortical area. Our findings agree with other studies on adult rodents, where an increased ultimate strength have been associated with exercised tibiae in 15 and 10 week old rats^31^ and mice^74^, respectively. Bone stiffness can be related to its morphology and cross-sectional geometry in growing rats^77^. More specifically, stiffness can be associated with the increase or decrease in cortical thickness (Ct.Th), total area (Tt.Ar) and cortical area (Ct.Ar)^78,79^. Ct.Ar has been increased significantly for both MI and HI groups (Table 2). Also, Ct.Th and Tt.Ar significantly increased for the HI group (Table 2). Hence, a strengthened diaphysis associated with an increased stiffness can be justifiable for the MI and HI groups. Increased ultimate load and stiffness was also observed in a study^31^ of 15-week old swimming and running rat groups along with an increased cortical area in the swimming groups. Another study with 10 week old mice reported an increased cortical thickness and stiffness, along with the cortical and total area increment in the loaded limbs^74^.

No effects were found on Young’s modulus when comparing LI, MI and HI loadings to shams (Table 3). Young’s modulus or bone rigidity is an intrinsic mechanical property of the bone^80,81^. With the greater structural strength found in the MI and HI groups (Table 3), an increase was also expected in the Young’s modulus. In a bending test, the evaluation of rigidity depends linearly on stiffness and cubicly on the span length of the tibiae (Equation 2)^82^. In the MI and HI groups, tibial lengths decreased by 4% and 5%, respectively. Even though their stiffnesses increased, the decreased span lengths in the cubic form might have counteracted this change, yielding unchanged Young’s moduli. Another study also reported non-significant changes in Young’s modulus in growing mice limbs^74^ undergoing 2 weeks of axial loading regime generating a maximum 2400 με at the mid-diaphysis of the tibiae.

Energy to failure load increased significantly for the MI and HI groups (Table 3). Greater energy to failure loads implies that the MI and HI tibiae sustained more deformation or strain before failure. Strengthening of bone tissue in the MI and HI groups is associated with its adaptation in response to the applied loading regime. During the loading period, compositional alterations might have occurred within the newly forming and pre-existing bone cellular matrix^83^, possibly involving type I collagen, which is known to affect the post-yield behavior of the bone^84,85^. Biochemical changes might have also interfered with collagen fibers orientation within the bone matrix and thus altered the toughening-mechanism in the bone microstructure^86^. Ultimate stress (σ_ult_) and failure stress (σ_fail_) are significantly increased and decreased respectively in both MI and HI groups (Table 3). The ultimate/failure load is the controlling factor in the corresponding ultimate/failure stress for the same group of tibiae (Equation 3)^31,87^. The respective significant increase and decrease in F_ult_ and F_fail_ for the MI and HI groups can explain the significant change in σ_ult_ and σ_fail_ for the same groups. Our results agree with other studies, where 15-week old rats exhibited increased stress and failure loads in the trained limbs^31,88^. Moreover, studies using 8-week old mice also observed increased ultimate stress in the loaded limbs after 2 weeks of axial loading regime^74^.

### Limitations

The current study has limitations involving some methodological aspects. For the trabecular VOI, only the proximal metaphysis was analyzed. It was chosen over the distal section because of the large amount of trabeculae in this region. Also, it has been reported that the proximal tibia has greater bone volume compared to the distal tibia^89^ and has also been used more often in bone remodeling studies^16,19,74^. As for bone growth rate, it was only measured at the proximal site. This choice was justified as proximal metaphysis is responsible for blood supply and vascular stasis in growing bone and the contribution of the proximal tibial growth plate in the total longitudinal growth was found to reach approximately 80% in adolescents^90^. So, any loading effects on proximal bony region could presumably be considered as to have the main effects between the proximal and distal parts as well. Also, the biological effects of bone remodeling were not investigated as part of this study. A future study could investigate bio-markers to infer on bone formation and resorption for better understanding of bone growth mechanism involved in impact loading regimes. Moreover, rats were sacrificed immediately after the last *in vivo* loading regime at 81 days old. A detraining period before the sacrifice, which might have modified the growth plate histomorphometry^47,91^, was not evaluated as our primary objective was to investigate the bone growth rate and histomorphometry while the growth plate was still active^92,93^.

## Conclusion

A significant decrease in body weight associated with a reduction in food intake was found for the high impact group. A thicker growth plate was observed for medium and high impact group despite having a significant longitudinal growth retardation. Also, the medium and high impact groups benefitted the trabecular and cortical microstructure and led to significant changes in structural- and tissue-level mechanical behavior in the diaphysis. The low impact group also altered bone structure, by exhibiting an increasing or decreasing trend in certain bone microstructural properties, but these changes were not statistically significant. In summary, a brief (10 min) daily exercise of medium (850 µε) and high (1250 µε) impact physical activity during adolescence can influence bone growth, and improve the quality and mechanics of bone microstructure at the end of the growing period. This loading model provides the scope to fully understand the role of controlled mechanical loading during the adolescent period and will be used in future for the design of non-invasive loading models to modify the bone microstructure and mechanical strength for building up a healthy and robust skeleton. Future work will investigate whether these impact loads applied during puberty are determinant factors for bone quality and strength in adult life.

## Materials and Methods

### Animals

Animals (n = 30, male, Sprague-Dawley rats) were obtained from Charles River Laboratories (Montreal, Canada) at approximately 3 weeks of age and allowed to acclimate for one week before the start of *in vivo* loading. Animal care and use conformed to the guidelines of the Canadian Council on Animal Care (CCAC), and the Institutional Animal Care Committee approved the experimental protocol at the Sainte-Justine University Hospital, Montreal, Canada. Rats were housed two per cage at 25 °C with a 12-h light/dark cycle. Standard laboratory diet and water ad libitum were provided. Three days prior to the beginning of loading, rats were randomly divided into five groups (n = 6/group): control, sham, low impact (LI), medium impact (MI), and high impact (HI). Body weights and food intakes were monitored weekly from the beginning (4^th^ week) of *in vivo* loading until the end (11^th^ week) to monitor overall health.

### *In vivo* axial tibial loading

**Figure 4:**
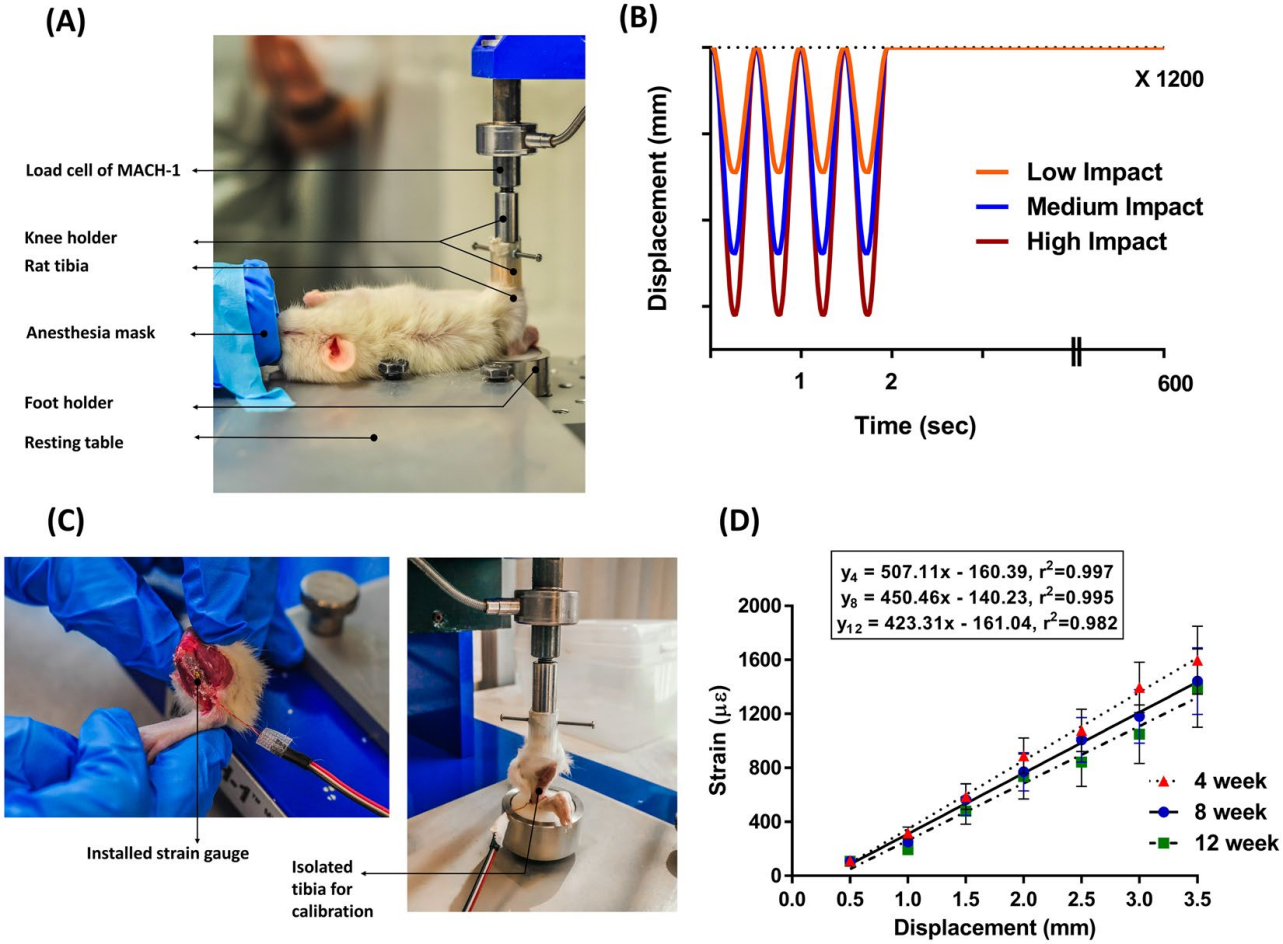
Impact loading setup and strain gauge calibration. (**A**) With the rats under anesthesia, the right tibiae from LI, MI and HI groups were loaded using a waveform generating 450, 850, and 1250 µε at the medio-proximal tibial surface from the 4 to 11 week period. (**B**) The loading profile consisted of haversine waveform displacements at 2 Hz and characterized by symmetric loading/unloading with a 0.10 sec of rest insertion between loading cycles. Loadings were repeated for 1200 cycles, yielding a daily (5 days/week) loading period of 10 minutes. (**C**) Strain gauge positioned at the medio-proximal surface of the tibia for allowing strain assessment for 0.5 mm to 3.5 mm of displacement. (**D**) Linear relationship between applied displacement and resulting strain at the medio-proximal surface of 4, 8 and 12 week old rat tibiae (mean value ± SD) (N = 6 rats/group).

While the rats of the LI, MI, and HI groups were kept anesthetized (2% isoflurane, 1.0 L/min O_2_), the cyclic impact loading was applied to the right tibia with a custom-built impact loading device (Fig. 4A). The device was controlled using a Mach-1 V500C (Biomomentum Inc., Montreal, Canada). Haversine waveform displacements^94^ (derived from the calibration experiment) were applied at 2 Hz and characterized by symmetric active loading/unloading with a 0.10 sec of rest insertion between load cycles^48,95^ (Fig. 4B). The frequency of 2 Hz was selected as it matches with the stride frequency range observed in rat normal locomotion^48^. A compressive preload of 0.5 N was applied to keep the tibia in position. Loadings were repeated for 1200 cycles, yielding a daily (5 days/week) loading period of 10 min^16^ (Fig. 4B). Sham rats received the same experimental setup conditions without any load application. Controls were kept in the cages without any manipulation. Regular cage activity was allowed between loading sessions.

### Strain calibrated impact loading

Prior to implementing the loading experiments, impact parameters were strain calibrated. The relationship between applied displacement and bone tissue deformation for the right tibia was established in an *in vivo* compression-strain calibration experiment^94^ for the 4, 8 and 12-week old rats. This relationship was adapted for determining the required displacement to generate 450, 850, and 1250 µε at the medio proximal surface of the rat tibia^96–99^. These strain magnitudes were chosen to correspond to peak strain values in the human tibia during unrestricted walking (450 µε), zig-zag uphill running (850 µε), and vertical jumping (1250 µε) conditions^96–99^. Animals (n = 18, male, Sprague-Dawley rats) were obtained from Charles River Laboratories (Montreal, Canada) at approximately 3, 7 and 11 weeks of age (n = 6/age group) and allowed to acclimate for one week before the strain calibration experiment. After CO_2_ asphyxiation, followed by decapitation, right tibiae were collected for each age group of rats. Tibiae were isolated from the middle of the femur to the toes. After tibial collection, an incision was made near the medio proximal surface of the tibia. Overlying skin and muscles were retracted to expose the bonding surface, polished with an abrasive paper and cleaned with ethanol solution. A single element strain gauge (C2A-06–015LW-120; dimensions: 0.86 mm × 1.32 mm, Micro-Measurements Group, Raleigh, NC, USA) was bonded with cyanoacrylate (M-Bond 200; Micro-Measurements Group) at 35% of the tibial length (L) (Fig. 4C). A compressive preload of 0.5 N was applied to keep the tibia in position (Fig. 4C). Haversine waveform displacements were applied at 2 Hz with a 0.10-sec rest insertion between displacement cycles. Displacements ranging from 0.5 mm to 3.5 mm were applied with a 0.50 mm increment. The strain data were recorded simultaneously at 2.5 kHz with a PC via Labview software (Labview 8.6, NI) through the quarter-bridge completion and analog input modules. Applied displacement and resulting strain were also recorded simultaneously. Displacement versus strain curves was plotted for three age groups, and a linear fit was applied to obtain the compression-strain relationships (Fig. 4D). Using these calibration curves, the amount of axial displacements were determined for producing the target tensile strain (450, 850, and 1250 µε) in LI, MI, and HI group of rat tibiae, respectively. The axial displacement values for the week’s in-between the chosen calibration studies were adjusted by linear interpolation using the two known displacement values and the weekly-age of rats.

### Micro computed tomography (micro-CT)

#### Weekly scanning regime

A micro-CT scanner was used to perform eight weekly CT scans of the right tibia of the rats, from 4 to 11 weeks of age. The imaging system was a Skyscan 1176 *in-vivo* micro-CT (Skyscan, N.V., Belgium) scanner with rotatable X-ray source and detector. Following the weekly loading regime, rats were anesthetized (2% isoflurane, 1.0 L/min O_2_), positioned on the carbon fiber half-tube bed of the Skyscan 1176, and maintained on anesthetic gasses for the duration of the scanning process (5–12 min). A cylindrical shape Styrofoam holder was used to position the right tibia to ensure the placement of the tibia along the scanner midline. All scans were performed with 18 μm voxel resolution, 65 kV, 384 μA, 350 ms exposure time, 0.65° rotation step, and a 1-mm Al filter^93^. A Phantom calibration was performed for each scanning day, prior to bone imaging using two cylindrical hydroxyapatite phantoms (0.25 and 0.75 g/cm^3^ CaHA). Images were reconstructed using NRecon software (v.1.6.10, Bruker-microCT, Belgium)^93^.

#### Trabecular bone morphometry

**Figure 5:**
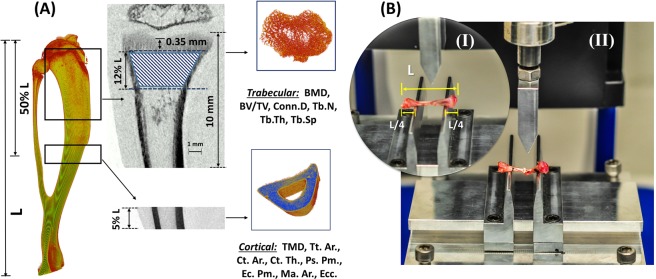
Trabecular and cortical volume of interests and experimental setup for three-point bending tests. (**A**) Representative 3D reconstructed tibia showing the total tibial length (L). The trabecular VOI started at ~0.35 mm distal to the growth plate and extended for 12% of the overall bone length (L). The VOI for cortical bone was centered at the tibial mid-diaphysis and extended proximally and distally for 5% of the tibial length (L). Volumes of interest including only trabecular and cortical bone were semi-automatically segmented using an in-house algorithm. (**B**) *I*-Experimental setup for the three-point bending tests. A distance of 50% of the total tibial length was fixed between the supports, while the remaining 50% was distributed equally between the external sides of the supports. *II*- Representative image of the fractured tibia after the bending test.

The volume of interest (VOI) for trabecular bone was selected as a percentage of the entire tibial length (L) (Fig. 5A). The VOI started at ~0.35 mm distal to the growth plate, excluding the primary spongiosa, and extended for 12% of the total tibial length (L)^19,93^ (Fig. 5A). The volumes of interest, including only trabecular bone, were semi-automatically segmented using an in-house algorithm^93^. For all analyses, a global gray threshold value of 65 corresponding to an equivalent density of 0.413 g/cm^3^ of calcium hydroxyapatite (CaHA) was applied^19,93^. CTAn software v.1.16 was used for performing morphometric analysis for the selected VOIs of trabecular bone and allowed evaluating the following parameters: bone mineral density (BMD), bone volume fraction (BV/TV), trabecular thickness (Tb.Th), trabecular number (Tb.N), trabecular spacing (Tb.Sp), and connectivity density (Conn.Dn)^100^ (Fig. 5A).

#### Cortical bone morphometry

The VOI for cortical bone was chosen from the tibial mid-diaphysis and extended proximal-distally for a total of 5% of the tibial length (Fig. 5A). The volumes of interest, including only cortical bone, were semi-automatically segmented using an in-house algorithm^93^. A global gray threshold value of 65 was set for all analyses^19,93^. Cortical microarchitectural measurements, including tissue mineral density (TMD), cortical bone area (Ct.Ar), total area (Tt.Ar), medullary area (Ma.Ar), cortical thickness (Ct.Th), endocortical perimeter (Ec.Pm), periosteum perimeter (Ps.Pm), and mean eccentricity (Ecc) were evaluated from the cortical bone VOIs^100^ (Fig. 5A).

### Mechanical testing

Following the last micro-CT scan (11^th^ week), rats were sacrificed using CO_2_ asphyxiation, followed by decapitation. Right tibiae (n = 30) from all groups of rats were cleaned of soft tissues and tested to failure in three-point bending under displacement control at 0.20 mm/sec using a Mach-1 V500C (Biomomentum Inc, Montreal, Canada). A custom made bending setup was used to place the rat tibiae horizontally with the anterior surface upwards and centered between the supports (Fig. 5B). A distance of 50% of the total tibial length was set between the supports, while the remaining 50% was distributed equally between the external sides of the supports (Fig. 5B). The structural properties of the tibial samples, including the ultimate load (N), failure load (N), energy to ultimate load (mJ), energy to failure load (mJ), and linear stiffness (N/mm) were determined using force-deformation curves. Energies to ultimate load and failure load were computed as the areas under the force-deformation curves. Stiffness was calculated as the slope of the linear portion of the force-deformation curve. To calculate the intrinsic cortical mechanical properties, cross-sectional parameters were measured using the micro-CT images at the tibial mid-diaphysis^87^. Assuming the tibial cross sections were elliptically shaped^101^, the moment of inertia was evaluated using the following equation:

1 $$I=\frac{\pi [a{b}^{3}-(a-2t){(b-2t)}^{3}]}{64}$$

where *I* is the moment of inertia (mm^4^), a is the width of the bone cross-section in the mediolateral direction (mm), b is the width of the bone cross-section in the anteroposterior direction (mm), and t is the average of the cortical thickness (mm)^31^. Assuming linear elastic bone material^82,102,103^ and using the beam bending theory, Young’s modulus and ultimate stress were determined by the following equations:

2 $$E=\frac{k{L}^{3}}{48I}$$

3 $${\sigma }_{ult}=\frac{{F}_{ult}Lc}{4I}$$

where, E is the Young’s modulus (GPa), and σ_ult_ is the ultimate stress (MPa), *k* is the stiffness (N.mm^−1^), L is the span length (mm), F_ult_ is the ultimate load (N), and c is the distance from the cross-section centroid to outermost point on the cross-section (mm), which was approximated as bone width/2^87^. Moreover, energies to ultimate stress (mJ/mm^3^) and failure stress (mJ/mm^3^) were assessed by calculating the respective areas under the stress-strain curve.

### Bone growth rate assessment

#### Calcein injections

Calcein was used to mark the newly formed bone line on the proximal tibial surface to enable longitudinal bone growth rate measurement. Injections of calcein (Sigma-Aldrich, St. Louis, MO, USA) were made intraperitoneally at a dosage of 15 mg/Kg^44^ at 5 and 2 days prior to euthanasia.

#### Tissue processing

Proximal sections (~10 mm) from each tibia were fixed for 48 h using the formalin solution (Anachemia, Montreal, QC, Canada). For dehydration and clarification, graded alcohol solutions and xylene were used respectively. Embedding process was performed using methylmethacrylate (MMA) (Fisher Scientific Canada, Nepean, ON, Canada)^43^. After polymerization, tibial blocks were cut in 6 µm sections using a microtome (Leica SM2500). The longitudinal bone axis was used to cut the tibiae for 36 slides, six series of six slides, containing two sections per slide. For each tibia, the first slide of each series was set aside from light for growth rate measurements, for a total of 6 slides (i.e., 12 sections total). Microscopic observations were performed with a microscope (Leica DMR with Retina Qimaging Camera) using 2.5x magnification.

#### Growth rate calculation

Bone growth rate was calculated as the distance between two calcein labels divided by the time interval (3 days) between injections^104^ (Fig. 2A). Measurements were performed using a custom-made Matlab program, where both calcein lines were modeled as splines, and the distance between the labels was automatically calculated as the mean value of 100 segments parallel to the longitudinal growth direction^43,44^ (Fig. 2A).

### Growth plate histomorphometry

Growth plate histomorphometric parameters were assessed to infer on the effects of impact loadings, similar to previous studies on bone growth mechanobiology^42,43^. These parameters included the PZ and HZ heights, hypertrophic cell height, as well as the number of proliferative cells per column^43,44^ (Fig. 3A,B). Zone heights and hypertrophic cell height were measured by implementing a 10x and 20x magnified image sets, respectively, following a similar approach to bone growth rate measurement. Average zonal height values were calculated from 100 individual segmental measurements (Fig. 3A). Also, 20x magnified image sets were used for measuring the number of proliferative chondrocytes per column (Fig. 3B). For each proximal tibial segment, histomorphometric parameters were measured by averaging 72 values, 6 values per section, and 12 values per microscope slide with a six series repetition.

### Statistical analysis

SPSS Statistics (v. 23, IBM) was used to perform statistical analyses. To determine time effects, loading effects, and their interaction on body weight and food consumption during the experimental period, a repeated ANOVA test (general linear model) was performed. The sham and control groups were compared to isolate any handling and manipulation effects, whereas, the impact groups were compared with the sham group to infer on the impact loading effects. For assessing any significant differences in average bone growth rates and histomorphometric parameters measured at the 11^th^ week for all tibiae, a paired Student’s t-test was implemented. Moreover, the structural properties of trabecular and cortical bone microstructure from all rat groups were statistically analyzed after 4 and 8 weeks of experimental period. Mechanical properties extracted from the three-point bending tests were also statistically analyzed. A one-way ANOVA with Tukey’s multiple comparisons was performed to assess the significant group difference and pairwise comparisons. Data are presented as means ± s.d. Statistical significance was fixed at *p* < 0.05.

## Supplementary information

Measurement process of bone growth rate using calcein image
