# Supplementary material for: High Impact Exercise Improves Bone Microstructure and Strength in Growing Rats
Source: Sci Rep. 2019 Sep 11;9:13128. doi: 10.1038/s41598-019-49432-2 (PMC6739374; doi:10.1038/s41598-019-49432-2)

**High Impact Exercise Improves Bone Microstructure and Strength in Growing Rats**

Tanvir Mustafy^1,2^, Irène Londono^2^, Florina Moldovan^2,3^, Isabelle Villemure^1,2^*

^1^ Department of Mechanical Engineering, École Polytechnique of Montréal, P.O. Box 6079, Station Centre-Ville, Montréal, Québec, Canada H3C 3A7.

^2^ Sainte-Justine University Hospital Center, 3175 Côte-Sainte-Catherine Rd., Montréal, Québec, Canada H3T 1C5.

^3^ Department of Stomatology, Faculty of Dentistry, Université de Montréal, Montreal, P.O. Box 6128, Station Centre-Ville, Montréal, Québec, Canada H3C 3J7.

* Corresponding author

Email: [isabelle.villemure@polymtl.ca](mailto:isabelle.villemure@polymtl.ca) (IV)

Figure A1: Calculation process of bone growth rate using custom made Matlab program


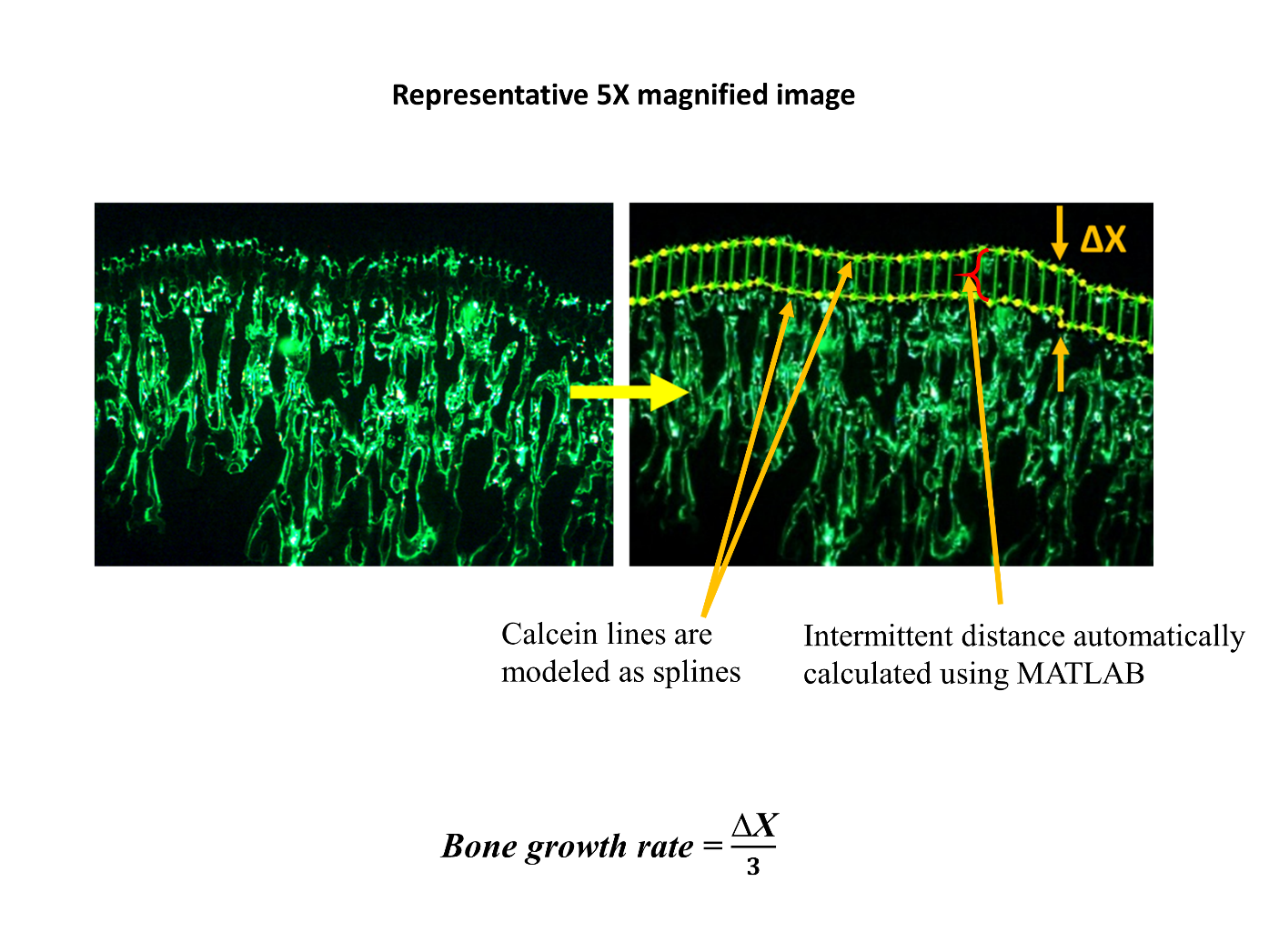

Supplement: Supplementary file 1 — Measurement process of bone growth rate using calcein image [file 41598_2019_49432_MOESM1_ESM.docx]
